# Recent Advances in the Observation and Modeling of Aerosol-Cloud Interactions, Cloud Feedbacks, and Earth’s Energy Imbalance: A Review

**DOI:** 10.1007/s40726-025-00382-6

**Published:** 2025-08-18

**Authors:** Takuro Michibata, Casey J. Wall, Nagio Hirota, Brandon M. Duran, Toru Nozawa

**Affiliations:** 1 Research Institute for Applied Mechanics, Kyushu University, Fukuoka, Japan; 2 Department of Meteorology, Stockholm University, Stockholm, Sweden; 3 Earth System Division, National Institute for Environmental Studies, Tsukuba, Japan; 4 Scripps Institution of Oceanography, University of California, San Diego, CA USA; 5 Department of Earth Science, Okayama University, Okayama, Japan

**Keywords:** Aerosol–cloud interactions, Cloud feedback, Radiative forcing, Earth’s energy imbalance

## Abstract

**Purpose of review:**

This review focuses on improving the understanding of the effects of anthropogenic aerosols on cloud processes, precipitation, radiation, climate, associated feedback mechanisms, and Earth’s energy imbalance (EEI), with a particular emphasis on literature published after the IPCC AR6.

**Recent findings:**

EarthCARE, an epoch-making satellite mission, has just been launched in 2024. Global climate models (GCMs) have become sophisticated, particularly with respect to the treatment of precipitation, and novel GCMs have been developed for the upcoming Coupled Model Intercomparison Project (CMIP). Satellite simulators have been used to incorporate new diagnostics to facilitate an apples-to-apples comparison between the models and observations established in the recent studies.

**Summary:**

To reduce the key uncertainties at fundamental process levels, we focus on: (1) assessing model-observation discrepancies, (2) improving the existing models, and (3) examining the linkage between effective radiative forcing, cloud feedback, and the recent EEI trends.

## Introduction

Aerosols and clouds are key factors influencing the Earth’s climate system. Aerosols can be emitted directly from both natural sources and anthropogenic activities. They are commonly classified into sea salt, mineral dust, sulfate, black carbon, and organic carbon, each of which influences cloud microphysics and radiative properties in distinct ways.

Increased anthropogenic aerosols reflect more solar radiation back to space in cloud-free skies, compared with pre-industrial (PI) conditions, thereby causing a negative radiative forcing and cooling the climate. This cooling effect due to scattering aerosols (e.g., sulfate) is partly offset by an increase in anthropogenic absorbing aerosols (e.g., black carbon). The direct interactions between aerosols and radiation in cloud-free skies are referred to as aerosol-radiation interactions (ARIs). Perturbing aerosols simultaneously impact cloud formation by serving as cloud condensation nuclei (CCN), resulting in smaller droplet sizes, larger cloud albedo, and potentially longer cloud lifetimes. This process is called the aerosol-cloud interactions (ACIs), which also contributes to net negative radiative forcing. The Intergovernmental Panel on Climate Change Sixth Assessment Report (IPCC AR6) estimated the magnitude of the effective aerosol radiative forcing (ERFari+aci) from the PI era to PD (1750–2014) to be $-$1.3 $\mathrm {W\,m^{-2}}$, offsetting approximately one-third of the warming (positive radiative forcing) caused by anthropogenic greenhouse gas (GHG) emissions [1]. However, the estimated ERFari+aci includes large uncertainty, varying from $-$2.0 to $-$0.6 $\mathrm {W\,m^{-2}}$ (90% confidence interval; “very likely” hereinafter).

**Fig. 1:**
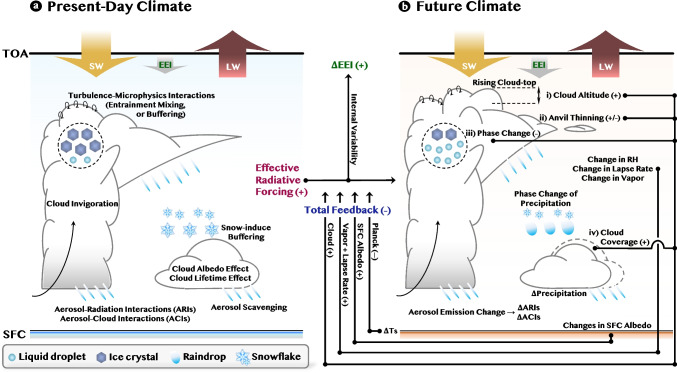
Schematic illustration of the relationships among ARIs, ACIs, adjustment processes, ERF, cloud and other feedbacks, and EEI. The symbol $\Delta$ represents the difference between two climate states: **a** present-day and **b** future climate. The four major cloud feedbacks are labeled as (i), (ii), (iii), and (iv) in **b**. The sign of the forcing or feedback is indicated as + or − in the figure when known. If the sign is unknown or debated, it is shown as ±-. Abbreviations: RH, relative humidity; EEI, Earth’s energy imbalance; SFC, surface; TOA, top-of-the-atmosphere; ARIs, aerosol-radiation interaction; ACIs, aerosol-cloud interactions; ERF, effective radiative forcing; SW, shortwave radiation; LW, longwave radiation

The key uncertainties in ACIs arise from the complex interplay between cloud microphysical and dynamical processes (Fig. 1), which is comprehensively discussed in the “Aerosol-cloud-radiation Interactions and their Uncertainties” section. Notably, ACIs depend strongly on the cloud types [2–4], regional environmental regimes [5–8], vertical inhomogeneities of aerosols and dynamics [9, 10], and their diurnal cycles [11]. Consequently, the magnitude of ERFaci exhibits regional [12] and seasonal [13] variability. However, global climate models (GCMs) do not adequately capture the regime-dependent behavior of ACIs owing to insufficient resolutions [13, 14], overly simplified parameterizations [15, 16], and the lack of microphysics-dynamics interactions [17], i.e., the two-way coupling between cloud microphysical processes and atmospheric dynamics (e.g., turbulent mixing and entrainment-evaporation feedback; [18]. Furthermore, ACIs depend on the cloud phase (i.e., liquid, mixed, and ice). Mixed- and ice-phase ACIs are more complex than liquid-phase ACIs [19, 20], and precipitation can buffer the cloud response to perturbed aerosols [21]. Therefore, aerosols influence cloud processes and precipitation formation. Conversely, precipitation can also modulate clouds and aerosols through the collision-coalescence of droplets and wet-removal processes [22, 23]. However, this bidirectional aerosol-precipitation interaction remains insufficiently understood.

For reliable climate prediction, complex ACI behaviors must be untangled, and cloud feedback processes must also be properly understood. Cloud feedback is a fundamental metric that quantifies the response of cloud radiative effects (CREs) to a unit temperature change caused by an external climate perturbation, typically increasing the $\mathrm {CO_{2}}$ forcing. The IPCC AR6 assessed the cloud feedback to be +0.42 $\mathrm {W\,m^{-2}\,K^{-1}}$ with large uncertainty, varying from $-$0.10 to +0.94 $\mathrm {W\,m^{-2}\,K^{-1}}$ (very likely) [1]. The major components of cloud feedback include: (1) positive high-cloud altitude feedback, (2) positive low-cloud amount feedback, (3) negative cloud phase feedback, and (4) anvil cloud area feedback. Although accurate representations require reasonable horizontal and vertical structures of clouds, along with the realistic classification of hydrometeor types (i.e., cloud droplet, ice crystal, raindrop, and snowflake), some systematic biases related to cloud coverage [24, 25], cloud- and precipitation-phase partitioning [26–30], and occurrence and intensity of precipitation [31] still remain, particularly in GCMs.

**Fig. 2:**
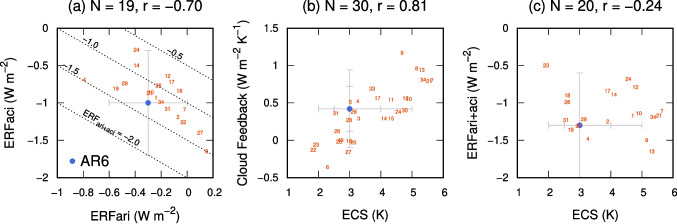
Relationship between **a** ERFari and ERFaci, **b** equilibrium climate sensitivity (ECS) and the net cloud feedback, and **c** ECS and ERFari+aci from the Coupled Model Intercomparison Project (CMIP6) models. Each subfigure denotes the number of models (*N*) in the plot, as well as the correlation coefficient (*r*). Blue dots with error bars denote the assessments from the Intergovernmental Panel on Climate Change Sixth Assessment Report (IPCC AR6). Error bars indicate the “very likely” range (> 90%), and the inner error bars in **b** and **c** indicate the “likely” range (> 66%). The dashed lines in subfigure **a** denote the contours of ERFari+aci. All data were acquired from Meehl et al. [33], Zelinka et al. [40, 51], Forster et al. [1], and Kuma et al. [52]; the plot numbers correspond to the model numbers and name listed in Table 1

However, recent model developments have addressed such commonly known biases, contributing to a better understanding of cloud feedback and ACIs [32–35], thereby enhancing the understanding of equilibrium climate sensitivity (ECS) at a fundamental process level [36–39]. Notably, the large spread in ECS among CMIP6 models is primarily attributable to differences in the representation of cloud processes across models [33, 40, 41]. Global satellite observations, essential for model development, have also continued to advance. The key components of the climate system have been monitored through long-term satellite records. The Clouds and the Earth’s Radiant Energy System (CERES) has been observing global cloud and radiation for almost 25 years (since 2000); active radar-lidar measurements from CloudSat and CALIPSO have been operating for nearly 20 years (since 2006); and the Global Precipitation Measurement (GPM) dual-frequency precipitation radar (DPR) has been in operation for over 10 years (since 2014). Additionally, the EarthCARE mission was just launched in 2024. With its novel capabilities, including the Doppler cloud profiling radar and the 355 nm wavelength high-spectral-resolution lidar, EarthCARE will provide a new insight into aerosol–cloud–radiation research [42–44].

Motivated by the substantial advances over the past decade, this review broadly summarizes the recent key findings on ACIs (“Aerosol-cloud-radiation Interactions and their Uncertainties” section) and cloud feedback processes (“Cloud Feedbacks and their Uncertainties” section), to address the following questions: (1) Where do the fundamental uncertainties come from? (2) What should be done to improve models? (“Advances in Precipitation Modeling in GCMs” section); and (3) How are ARIs and ACIs linked to the recent variations in the global energy budget from both observational and modeling perspectives? (“Interlinking Earth’s Energy Imbalance, ACIs, and Climate Feedbacks” section). The challenges and limitations in the field are briefly discussed in the “Future Directions and Ongoing Challenges” section, and the concluding remarks are presented in the “Conclusions” section.

## Aerosol-cloud-radiation Interactions and their Uncertainties

The IPCC AR6 assessed the anthropogenic aerosol forcing, ERFari+aci, to be $-$1.3 [$-$2.0 to $-$0.6 (very likely)] $\mathrm {W\,m^{-2}}$ [1]. This forcing is composed of ERFari of $-$0.3 [$-$0.6 to 0.0 (very likely)] $\mathrm {W\,m^{-2}}$ and ERFaci of $-$1.0 [$-$1.7 to $-$0.3 (very likely)] $\mathrm {W\,m^{-2}}$, with the latter depicting higher uncertainty (Fig. 2a, Table 1). An accurate estimation of the magnitude of aerosol forcing is urgently needed because future aerosol reduction driven by improvements in air quality and more stringent regulations on pollutants is likely to weaken ERFari+aci [45]. This weakening has direct implications for climate warming, as it reflects how changes in aerosol concentration influence the energy balance [46–48] and may further lead to changes in the intensity and frequency of precipitation [49, 50].

**Table 1:** Summary of the ERFari, ERFaci, ERFari+aci, ECS, and net cloud feedback, from the available 34 CMIP6 models [53]

	Model name	ERFari	ERFaci	ERFari+aci	ECS	Cloud Feedback
1	ACCESS-CM2	$-$0.24	$-$0.93	$-$1.17	4.7	
2	ACCESS-ESM1-5	$-$0.07	$-$1.19	$-$1.25	3.9	
3	AWI-ESM-1-1-LR				3.29	0.29
4	BCC-ESM1	$-$0.79	$-$0.69	$-$1.48	3.26	0.52
5	BCC-CSM2-MR				3.02	0.51
6	CAMS-CSM1-0				2.29	$-$0.36
7	CanESM5	$-$0.02	$-$1.09	$-$1.11	5.64	0.8
8	CESM2	0.15	$-$1.65	$-$1.5	5.15	0.96
9	CESM2-WACCM				4.68	1.17
10	CNRM-CM6-1	$-$0.28	$-$0.86	$-$1.14	4.9	0.55
11	CNRM-CM6-1-HR				4.33	0.54
12	CNRM-ESM2-1	$-$0.15	$-$0.64	$-$0.79	4.79	0.56
13	E3SM-1-0			$-$1.65	5.31	0.94
14	EC-Earth3	$-$0.39	$-$0.5	$-$0.89	4.1	0.29
15	EC-Earth3-Veg				4.33	0.29
16	FGOALS-f3-L				2.98	$-$0.01
17	GFDL-CM4	$-$0.12	$-$0.72	$-$0.84	3.89	0.56
18	GFDL-ESM4	$-$0.06	$-$0.84	$-$0.9	2.6	
19	GISS-E2-1-G	$-$0.55	$-$0.81	$-$1.36	2.71	0.0
20	GISS-E2-1-H				3.12	$-$0.03
21	HadGEM3-GC31-LL	$-$0.29	$-$0.87	$-$1.17	5.55	0.79
22	INM-CM4-8				1.83	$-$0.13
23	INM-CM5-0			$-$0.5	1.92	$-$0.06
24	IPSL-CM6A-LR	$-$0.39	$-$0.29	$-$0.68	4.56	0.38
25	MIROC-ES2L				2.66	$-$0.02
26	MIROC6	$-$0.22	$-$0.77	$-$0.99	2.6	0.12
27	MPI-ESM1-2-HAM	0.1	$-$1.4	$-$1.31	2.96	$-$0.16
28	MPI-ESM1-2-HR				2.98	0.27
29	MRI-ESM2-0	$-$0.48	$-$0.74	$-$1.22	3.13	0.38
30	NESM3				4.77	0.4
31	NorESM2-LM	$-$0.15	$-$1.08	$-$1.23	2.56	0.36
32	NorESM2-MM	$-$0.03	$-$1.26	$-$1.29		
33	SAM0-UNICON				3.72	0.69
34	UKESM1-0-LL	$-$0.2	$-$0.99	$-$1.19	5.36	0.81

Units of ERFs, ECS, and cloud feedback are $\mathrm {W\,m^{-2}}$, $\textrm{K}$, and $\mathrm {W\,m^{-2}\,K^{-1}}$, respectively. The data were sourced from Meehl et al. [33], Zelinka et al. [40, 51], Forster et al. [1], and Kuma et al. [52]. The model numbers and names correspond to the plots shown in Fig. 2

A key uncertainty arises from the regime-dependence of cloud response to aerosol perturbations [54–56]. In addition to the cloud albedo and lifetime effects, various adjustment processes, including *buffering* [57], can dampen the magnitude of ACIs [58, 59]. Notably, the microphysics of mixed- and ice-phase clouds are more complex than that of liquid-phase clouds [60, 61]; therefore, modeling the different dependencies of ACIs on the cloud phase and various aerosol types is a challenging issue in GCMs [16, 62].

**Fig. 3:**
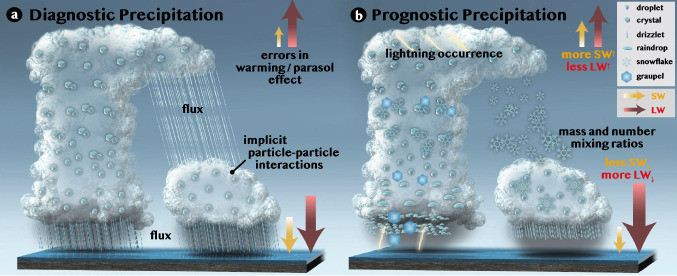
Schematic illustration of different treatment of precipitation with **a** diagnostic and **b** prognostic frameworks. Precipitating hydrometeors in the diagnostic scheme are represented as fluxes. Prognostic precipitation can keep its memory across multiple time-steps as mass- and number-mixing ratios, enabling explicit interactions among cloud and precipitation, and accounting for the radiative effects of precipitation (see text for details)

For the liquid phase, particularly stratiform low-level clouds under stable conditions, an increasing aerosol concentration enhances cloud water content, depicting the lifetime effect, though the magnitude varies across observation sources and regions [2, 6, 63–65]. However, cloud water content can decrease in response to increased aerosols, particularly under an unstable environmental regime, i.e., in regions dominated by cumulus clouds over the tropics and subtropics [66]. In non-precipitating clouds, decreased cloud water content may occur owing to increased aerosols [12], and this may be attributed to enhanced evaporation induced by entrainment mixing in an unstable regime. As smaller droplets tend to evaporate faster than larger droplets, cloud-top entrainment can efficiently evaporate droplets, intensifying cooling and resulting in stronger mixing. This leads to the formation of an entrainment-evaporation feedback loop [67]. Decreased cloud water content has been reported in previous observational studies [5, 12, 68] and in case studies of cumulus clouds in large-eddy simulations [69], as an effect of adjustment processes known as “*buffering mechanisms*” (Fig. 1) [57]. Such a bidirectional cloud water response in observations and process-modeling is in stark contrast to GCMs, which depict a coherent increased liquid water response to aerosol perturbations, as most GCMs primarily account for cloud lifetime effects [35, 70]. This emphasizes the importance of microphysics-dynamics interactions [17] for better simulations of ACIs in climate systems [71].

For deep convective clouds, in-depth discussions, particularly regarding the validity of the cloud-invigoration effect, have been presented in previous studies [72–74]. A popular hypothesis is that increasing aerosol concentrations enhance condensation below the freezing level and elevate more cloud droplets above it in convective clouds. This, in turn, changes the buoyancy and updraft speed of rising air parcels in the cloud [75]. However, this cloud invigoration effect is difficult to test with satellite data alone, and the magnitude in real clouds is controversial [76]. For mixed- and ice-phase clouds, two opposing cloud responses have been identified: (1) enhanced precipitation efficiency under increased ice nuclei (IN) with fixed CCN, referred to as the “glaciation indirect effect” and (2) reduced snowfall under increased CCN with fixed IN, referred to as the “riming indirect effect” [16, 60, 62]. These clouds, with particularly low cloud-top temperatures, are sensitive to both shortwave (SW) and longwave (LW) radiation. The treatment of mixed-phase clouds in models can be a major source of diversity in the ERFaci for both the SW and LW components [77], emphasizing the importance of cloud-phase partitioning for accurate ACI representation (Fig. 1).

Furthermore, precipitating hydrometeors can influence the magnitude of ACIs. The precipitation from upper clouds can fall into lower-lying clouds, wherein the descending ice or snow triggers precipitation in the lower clouds—-a process known as the “seeder-feeder” mechanism [78]. This process can effectively deplete the lower liquid clouds through riming and snow aggregation, thereby weakening the magnitude of ACIs [21]. Although the seeder-feeder-like mechanisms (“snow-induced buffering” hypothesis) are expected to occur specifically in multi-layered mixed-phase clouds, most GCMs do not include this process owing to their diagnostic treatment of precipitation (discussed later in Fig. 3).

Notably, the evaluations of ERFari and ERFaci are sensitive to how “all-sky” scenes are defined and separated into “cloudy-sky” and “clear-sky” scenes in relation to aerosol conditions [79] and the distance from the clouds [80]. CRE is ideally assessed based on the difference between with- and without-clouds under aerosol-free “clean-sky” conditions to remove contamination from aerosol scattering and absorption. In addition, a non-cloudy pixel does not always denote a “pure” clear-sky; the vicinity of clouds can be affected even if they are non-cloudy, due to invisible thin clouds and undetected sub-pixel clouds or enhanced aerosol scattering caused by hygroscopic swelling under high humidity conditions [81]. This requires the definition of three regimes: (1) cloudy-sky, (2) cloud-influenced-clear-sky, and (3) far-field clear-sky [80]. In addition, given the non-negligible contribution of precipitating hydrometeors to radiation [82], quantifying the “precipitation radiative forcing” from observations and numerical models needs further research [83].

## Cloud Feedbacks and their Uncertainties

Global warming can alter the micro- and macrophysical characteristics of clouds. Considering the significant impact of clouds on LW and SW radiation, depending on their altitude, thickness, amount, and phase [84], the responses of clouds to global warming in the future may result in feedbacks on the initial warming perturbation (Fig. 1).

Future warming is likely to raise the tropopause, elevate high clouds, and enhance atmospheric warming by trapping more LW radiation (positive high-cloud altitude feedback; Fig. 1b-(i)). Surface warming increases the temperature of the planetary boundary layer and reduces the boundary layer capping inversion if the free tropospheric temperature remains fixed. Warming of the boundary layer also causes stronger latent heat release in cloud updrafts, which enhances in-cloud turbulence and entrainment of dry free-tropospheric air into the boundary layer. These effects reduce low-cloud amount, causing a positive radiative feedback (positive low-cloud amount feedback; Fig. 1b-(iv)). Although a negative feedback can occur in regions where the boundary layer inversion is strengthened owing to the sea surface temperature (SST) pattern effect, the changes in low-cloud amount are believed to cause a positive radiative feedback on average across tropical and subtropical oceans [85, 86].

In contrast to positive cloud feedbacks, warming can also induce negative cloud feedback through phase change from ice to liquid, as smaller droplets reflect SW more efficiently (negative cloud phase feedback; Fig. 1b-(iii)). However, this process remains highly uncertain in numerical models [29]. Furthermore, thermodynamic arguments predict fewer anvil clouds under the effects of surface warming (tropical anvil cloud area feedback; Fig. 1b-(ii)). Although this is evaluated as a negative feedback in the IPCC AR6 [1] and the World Climate Research Programme (WCRP) [85], recent studies indicate an almost neutral [87] or positive [88] feedback, as anvils can exhibit either a positive or negative CRE depending on their life cycle stage [89].

As a consequence of these contributions, cloud feedback is assessed to be +0.42 [+0.12 to +0.72 (likely; > 66% probability); $-$0.10 to +0.94 (very likely)] $\mathrm {W\,m^{-2}\,K^{-1}}$ in the IPCC AR6 [1]. Larger cloud feedback implies stronger warming; therefore, the magnitude of the cloud feedback strongly correlates with ECS [40, 90], which is assessed to be 3 [2.5 to 4 (likely); 2 to 5 (very likely)] K (Fig. 2b). Note here that a higher ECS values, such as those exceeding 4.5K, are not strongly supported by observational constraints based on historical temperature trends [38].

To narrow these uncertainties, the recognized uncertainties in cloud feedback (“known unknowns”) must be reduced, and the feedback mechanisms whose uncertainties remain entirely unknown (“unknown unknowns”) must be explored. Global warming is likely to increase the occurrence of liquid clouds through the aforementioned cloud phase feedback, which is also associated with uncertainty in the representation of the warm cloud lifetime effect in models. GCMs tend to precipitate faster than satellite-retrieved estimates [49, 91], potentially related to the underestimation of the “cloud lifetime feedback” [50].

The changes in cloud phase are generally related to the SW component; however, its effect on the LW component is also important, particularly for Arctic mixed-phase clouds. Owing to the lower precipitation efficiency of liquid clouds, compared with that of ice clouds, the replacement of ice with liquid would increase the cloud amount, thereby enhancing the downward LW radiation and resulting in increased surface warming [92]. Furthermore, under global warming, initially icy Arctic clouds with low emissivity are expected to undergo a phase shift toward liquid, resulting in higher emissivity due to the radiatively more opaque nature of liquid clouds. The so-called “LW cloud emissivity feedback” also provides positive feedback [93]. These positive feedbacks can strengthen Arctic amplification through LW contribution, which is in stark contrast to the general concept of the cloud phase feedback mechanism that is based on SW radiation.

Considering the difference between radiatively clear rain and radiatively opaque snow [94, 95], the precipitation phase partitioning is also linked to the changes in radiative forcing, through snow-to-rain phases transition under anthropogenic warming (precipitation phase feedback). This may potentially perturb the magnitude of the ECS [31]; however, this aspect requires further investigation (“unknown unknowns”).

Analyzing the potential relationship between ECS or cloud feedback and ERFari+aci (Fig. 2c) may offer a pathway to constrain these uncertainties. A negative correlation can be found in both multi-model variability [33] and single-model experiments [37]. In other words, the models compensated for a higher ECS by simulating a more negative ERFari+aci to reproduce a consistent historical temperature record [90]. The correlation between cloud feedback and aerosol forcing can be linked to the liquid water path (LWP). The models with higher LWP are more likely to be exposed to anthropogenic aerosol perturbations (more negative ERFaci) and experience fewer opportunities for ice-to-liquid phase transitions (less negative cloud feedback and higher ECS) [96]. As LWP is a widely observed parameter, it can serve as an effective constraint for better understanding model uncertainty [58, 96, 97].

Regarding ERFari+aci, a compensatory relationship between ERFari and ERFaci can be observed (Fig. 2a), wherein the models with less negative ERFari tend to exhibit more negative ERFaci. Although this strong negative correlation has not yet been thoroughly investigated, it may be linked to how models partition clear-sky and cloudy-sky regions, as the former primarily relates to ARIs, whereas the latter is associated with ACIs. Addressing the physical mechanisms behind the relationship is crucial for a better understanding of aerosol ERFs, cloud feedback, and ECS [37, 98, 99]. To reduce the uncertainties in climate predictions, constraining these processes from combined use of observable variables is a desirable approach [29, 50, 100].

The process-level evaluation can be conducted using a satellite instrument simulator [101]; note that this approach should be used for conducting “scale-aware” and “definition-aware” comparisons (i.e., apples-to-apples comparison) between models and satellite retrievals [102]. Recent developments in simulators, with respect to incorporating online diagnostics for precipitation processes [91, 103], aerosol forcings [35], cloud feedback, and ECS [39], can support process-oriented model evaluations. In this context, recent advancements in the EarthCARE simulator can serve as a powerful tool for maximizing the value of observational data [104–107].

## Advances in Precipitation Modeling in GCMs

A common goal in GCM development is to improve the physical basis of the model parameterizations to achieve more accurate weather and climate simulations [108, 109]. With respect to aerosol-cloud-precipitation interactions, one of the major bottlenecks in GCMs is the treatment of precipitation (Fig. 3). Recent advances in precipitation modeling have reduced systematic biases. This section provides an overview of how the replacement of diagnostic precipitation with prognostic precipitation can improve microphysical and macrophysical aspects, as well as the energy budget of the climate system.

Owing to the historical constraints on computing resources, the majority of GCMs rely on the diagnostic treatment of precipitation (Fig. 3a). This modeling framework assumes that precipitating hydrometeors are instantaneously removed from the atmosphere within a single model timestep as surface precipitation. This assumption holds true only when precipitation falls at a relatively fast speed ($\sim$5 $\mathrm {m\,s^{-1}}$), allowing a drop of 9 km during the typical 30-min timestep of traditional GCMs. However, the drizzles from the stratocumulus and snowflakes from high clouds fall at a much slower speed, violating this assumption. Additionally, with enhanced computational resources, simulations can now achieve higher spatial (tens of kilometers) and temporal (approximately 10 min) resolutions [53, 95]. In such cases, diagnostic precipitation may introduce inconsistencies because it does not retain the memory of the precipitating hydrometeors in the atmosphere and neglects the advection of precipitation. These limitations in diagnostic precipitation can lead to biases in excessive autoconversion [110], susceptibility to aerosol perturbations [12, 111], and excessive light rain [49, 112].

Accordingly, some GCMs incorporate a prognostic precipitation framework [95, 113–117]. This modeling framework can retain the memory of precipitating hydrometeors in the atmosphere across multiple timesteps, enabling explicit cloud-precipitation interactions (Fig. 3b). This approach can enhance accretion over autoconversion, which is in agreement with in situ observations [113]. Autoconversion is the only pathway through which aerosols can prolong cloud lifetime. In contrast, accretion depends only on cloud water and rainwater masses, and is independent of aerosol perturbations [118, 119]. Therefore, it can reduce cloud susceptibility to aerosols and weaken the magnitude of ERFaci [13, 95, 113, 120]. The accretion process depletes cloud droplets and provides more opportunities for the wet scavenging of aerosols, both of which weaken the ERFaci [23]. Notably, more realistic ERFaci and improved representation of warm rain formation can be simultaneously achieved by including prognostic precipitation, which cannot be achieved by traditional diagnostic precipitation owing to error compensation [49, 112].

A new adjustment process that weakens ERFaci was also identified with the introduction of prognostic precipitation. In this process, precipitating hydrometeors from high clouds trigger precipitation in lower-lying clouds, a phenomenon widely known in mesoscale meteorology as the “seeder-feeder” effect. This mechanism can also be crucial in climatology, as low clouds tend to be depleted through riming and aggregation by overlying high clouds, thereby weakening the ACIs. The “snow-induced buffering” hypothesis [21] suggests that this process occurs particularly in multi-layered mixed-phase clouds. However, traditional diagnostic precipitation models cannot capture this aspect because of their implicit treatment of cloud-precipitation interactions.

The explicit consideration of the radiative effect of precipitation (REP) is another important advantage in prognostic precipitation (Fig. 3). Similar to cloud droplets and ice crystals, raindrops and snowflakes reflect SW and also absorb and re-emit LW radiation. REP alters the vertical thermodynamic structure by inserting more heating into the atmosphere and enabling surface cooling. This effect leads to atmospheric stabilization and weakens the tropical circulation [121], atmosphere–ocean coupling [83], and lifecycle of El Niño-Southern Oscillation (ENSO) activity [90]. A global modeling survey of REP indicated that neglecting the atmospheric radiative cooling may slow down the hydrological cycle, resulting in a $\sim$4% decrease in the global mean precipitation, particularly over the tropics [82]. As the snow water path is more abundant, compared with that of cloud ice or rainwater, and is more concentrated over higher latitudes [21], the radiative effect of snowfall has a significant influence in polar regions [122, 123]. The LW warming effect by snowfall increases the polar surface temperature, particularly during the polar night season, by at least 1 K or more, which is potentially related to Arctic amplification [82]. The inclusion of REP in GCMs can improve the systematic bias in weaker Arctic warming in CMIP models [93, 124, 125].

Prognostic precipitation and REP can also influence cloud feedback and ECS. Sensitivity experiments conducted using GISS-ModelE3 and extensive analyses of the CMIP models have shown that the inclusion of REP increases the cloud feedback [126]. Although the increased ECS in CMIP6, compared with that in CMIP5, is thought to be partly driven by the greater supercooled liquid ratio [40], it is also partially attributable to the inclusion of REP, which is increasingly included in CMIP6 models [83]. Additional investigations by MIROC6 also increased cloud feedback, resulting in an increase in ECS of approximately 20% [34], attributable to the improved (i.e., increased) representation of the global cloud coverage. Interestingly, the stronger cloud feedback in MIROC6 can be largely attributed to the increase in the LW component of the cloud altitude feedback [34], even though previous studies indicated that a reduction in the SW negative cloud phase feedback was responsible for driving stronger cloud feedbacks [36, 126]. This difference needs further investigation when additional GCMs with prognostic precipitation and REP become available.

Despite significant recent advances in precipitation modeling, representing heavy, localized precipitation remains a common issue in GCMs. This problem seems to align with the fact that GCMs generally ignore larger ice hydrometeors (i.e., graupel and hail), which are sometimes important for severe convective storms. This requires the explicit implementation of aerosols or microphysics into convective parameterization [127, 128], with prognostic graupel/hail parameterization [129]. The Predicted Particle Properties (P3) scheme has succeeded in simulating realistic size distributions and physical ice-growth processes by treating various ice hydrometeors as a single category with a higher moment [130, 131], which is also a promising approach for extreme rain events [132, 133].

Graupel is non-negligible in polar climate [134]; it triggers lightning [135, 136], which may increase the risk of wildfire over the Arctic [137]. This can further accelerate future warming through additional $\mathrm {CO_{2}}$ and methane emissions from permafrost [138], contributing to a positive feedback, despite the large uncertainties [139, 140].

Finally, note that parameterizations have various tunable knobs, including parameter thresholds or lower and upper limits of the physical variables. The numerical ordering of different physics schemes and model timestep can also influence the model performance [141, 142]. Future studies must examine the model behavior in response to the “minor-looking treatment”, to carefully interpret whether the model results are physically meaningful or artificially induced [143].

## Interlinking Earth’s Energy Imbalance, ACIs, and Climate Feedbacks

Earth’s energy imbalance (EEI), a fundamental global climate indicator, is defined as the balance between the energy entering Earth and the energy from Earth escaping to space [144] (Fig. 1). This simple metric provides a clear representation of the climate state by incorporating ERF, along with surface temperature changes ($\Delta T_{s}$) and the associated feedback [1, 145]. A positive imbalance at the top-of-the-atmosphere (TOA) signifies that the energy entering the system is more than that exiting the system, resulting in heat accumulation and an increase in $T_{s}$ until the equilibrium is reestablished. EEI therefore quantifies the net heat uptake by the Earth system due to the current imbalance between incoming and outgoing energy. In other words, EEI reflects the portion of the radiative forcing that has not yet been compensated by surface warming [99, 144]. As the change in EEI can be attributed to the total ERF, radiative response, and internal variability ($\epsilon$), EEI anomaly can be described as follows:

1 $$\begin{aligned} \Delta EEI = \Delta ERF + \lambda \Delta T_{s} + \epsilon \end{aligned}$$

where $\lambda$ ($\mathrm {W\,m^{-2}\,K^{-1}}$) denotes the net total climate feedback parameter [146, 147].

EEI over the period 1971–2020 is estimated to be +0.48 ± 0.1 $\mathrm {W\,m^{-2}}$, based on the Earth heat inventory [148], with steady acceleration being observed over the last two decades [149, 150]. Recent variations in the EEI originate from aerosols and clouds and cannot be explained by internal variability alone [147]. Therefore, large uncertainties in ERFari+aci may lead to the misrepresentations of EEI and climate change, along with the effects of future aerosol reductions [45].

Generally, EEI has been reported as an all-sky value, but splitting it into clear- and cloudy-sky values can be helpful for easily interpreting whether the variations in aerosols or clouds (CRE) may contribute toward the dominant EEI trend [48]. Similarly, decomposing these scenes into SW and LW components can provide more insight on the source of EEI anomalies [150]. For example, CERES satellite data indicates that in clear-sky conditions, the EEI trend is primarily driven by increased SW radiation [151]. GCMs systematically underestimate the positive clear-sky EEI [152] and the negative cloudy-sky LW EEI [48], resulting in a seemingly consistent all-sky EEI. These studies emphasize the requirement for better modeling of aerosols and clouds and also imply the difficulties in representing the combined “pattern effect” owing to the spatial variations in SST [153–155] and aerosol emissions [156, 157].

Model-observation discrepancies in EEI need to be continually assessed and validated using the most-up-to-date aerosol emission records. This is important because EEI variations driven by changes in aerosols and cloud coverage, in addition to internal variability, have been closely linked to the record-breaking heat events in 2023 and 2024 [145, 158, 159]. The extreme warming in 2023 and 2024 reflects not just long-term anthropogenic trends but also the amplification due to regional climate patterns (e.g., ENSO), emphasizing the importance of future studies aimed at elucidating the causes, mechanisms, and timing of these events [87, 160, 161].

More specifically, recent observational and modeling studies suggest that the reduction in sulfur aerosol emissions from the shipping sector following the International Maritime Organization 2020 regulation (IMO2020) has altered cloud microphysical properties [162] and radiative forcing [163]. Although the magnitude of surface temperature response to IMO2020 remains under debate [164, 165] and air-sea interactions are also considered to play a critical role [166], EEI remains a valuable metric for monitoring the state of the climate system [144, 167].

In this context, CERESMIP [168] can be expected to shed light on the source of the uncertainty between observations and models. Using updated forcings aligned with the CERES record from 2000 to at least the end of 2021, the ongoing CERESMIP project aims to assess the ability of the state-of-the-art GCMs to capture observed EEI trends. Continuous long-term satellite observations are essential for monitoring EEI, and sustained efforts are thus needed to ensure ongoing model-observation intercomparisons [169]. The Regional Aerosol Model Intercomparison Project (RAMIP; [170]) has likewise provided important evidence that reductions in East Asian aerosol emissions since the 2010s may have contributed substantially to the observed acceleration in global surface warming [171].

## Future Directions and Ongoing Challenges

The recent advances in modeling and observations described in earlier sections have enhanced our knowledge of ACIs, cloud feedback, and the associated EEI. Nevertheless, a variety of challenging tasks remain for achieving accurate weather and climate simulations, as explained below:

1. *Understanding dependence of ACIs on aerosol types and cloud regimes:* The process-level understanding of how different aerosol types affect the magnitude of ACIs remains poor [172]. Advanced treatments of the aerosol mixing state and activation processes are still challenging issues, and the recent discovery that surface tension prevails over the solute effect in organic-influenced cloud droplet activation has significantly advanced our understanding of model uncertainties [173]. In terms of cloud regimes, the EarthCARE Doppler cloud profiling radar can provide novel insights into microphysics-dynamics interactions by sorting vertical air motion, which is a source of model diversity [71, 174].
2. *Quantitative estimates of ERFaci from ice-containing clouds:* Most previous estimates of ERFaci have focused heavily on liquid clouds, and therefore, the SW component. Quantitative estimates of ERFaci from ice-containing clouds are almost entirely absent, at least at global and regional scales, and with uncertainty estimates. Microphysical adjustments for mixed- and ice-phase clouds are considered only in a limited number of GCMs [77], which can lead to diversity in LW ERFaci [21, 51, 175]. Improving ice microphysical parameterizations is essential for obtaining a more complete understanding of climate forcing, feedback, and ECS.
3. *Quantifying precipitation-induced feedback processes:* Considering the non-negligible radiative effects of precipitating hydrometeors [82, 83], precipitation-induced feedback is inherent to the Earth’s climate system. Global warming will likely cause a phase change from radiatively opaque snowflakes to less opaque raindrops [34, 126], emphasizing the importance of precipitation phase partitioning [30, 31]. Extending the kernel method to precipitating hydrometeors by applying active satellite sensors, such as CloudSat, CALIPSO, and GPM/DPR, with instrument simulators could facilitate seamless evaluation of clouds and precipitation [176, 177].
4. *Separating EEI all-sky contribution into the clear- and cloudy-sky values:* Although the all-sky EEI trend is composed of clear- and cloudy-sky contributions [150], GCMs tend to underestimate clear-sky trends, which are instead compensated for by overestimated cloudy-sky trends [48, 152]. This misrepresentation denotes that the models will have uncertainties in EEI sensitivity, particularly for different aerosol concentrations and cloud coverages, owing to future aerosol reduction and/or global warming [168]. The misrepresentation of the SW and LW components should be improved to reduce the uncertainties in the evaluations of warming diurnal asymmetricity [178], elevation-dependent warming [179, 180], and seasonal warming trends [181, 182].
5. *Understanding atmosphere-ocean-cryosphere interactions:* Aerosols and clouds are not only atmospheric processes, but they also interact with the ocean, land, and cryosphere; thus, systematic model biases could be related to different components. Increased aerosol transport to polar regions may accelerate surface snow melting, owing to the aerosol-induced change in the surface albedo [183, 184]. However, most models cannot explicitly capture these processes. Owing to global warming, increased clouds and graupel over the Arctic can increase the occurrence of lightning [135, 140], thereby increasing fire risk that can enhance $\mathrm {CO_{2}}$ forcing [137]. Notably, the current models do not consider this feedback; hence, future model development requires efforts to include such missing, unknown physical processes and feedbacks, even with a simplified treatment.
6. *Constraining structural uncertainty and parametric uncertainty:* A multi-model intercomparison study is an effective and popular approach for examining the uncertainties in simulations. Multi-model uncertainties arise from different formulations of various physical processes (*structural uncertainty*). However, various tunable knobs also cause important uncertainties that may obstruct the physical behavior of models (*parametric uncertainty*), which should be examined within a single-model framework using a perturbed parameter ensemble (PPE) approach [97, 185–187]. Although the tuning parameters, such as upper and lower bounds or thresholds of parameters, are often overlooked, more detailed discussions and exploration of the “minor-looking treatment” in models [143] should be documented for the upcoming CMIP7.

## Conclusions

This review highlights the recent advances in the understanding of ACIs, cloud feedback, and the associated EEI in global climate modeling and observations (Fig. 1). Despite the significant progress, several challenges remain in narrowing the uncertainties in climate projection. Although ACIs, cloud feedback, and EEI have been studied separately, focusing on their relationship can provide an in-depth understanding of the sources of multi-model diversity (Fig. 2; Table 1).

New missions and technologies can enhance our fundamental understanding. The EarthCARE mission contributes value to the study of aerosols and clouds. The application of machine learning and graphics processing unit acceleration has significantly improved both computational efficiency and predictive performance [188–191]. Faster computations enable higher-resolution modeling, which in turn, improves model accuracy. However, simply increasing model resolution does not automatically reduce the uncertainties in physical processes. In addition to GCMs, the insights from process modeling should be leveraged, e.g., cloud-resolving models and large-eddy simulations, along with the combined use of multi-platform satellite and in situ observations. This approach is essential for addressing the uncertainties in the physical processes of GCMs at a fundamental level (Fig. 3). Until we have a solid understanding of this process, the confidence in simulating realistic climate models remains limited.

## Data Availability

No datasets were generated or analysed during the current study.

## Code Availability

The processed data and scripts used to produce the figures can be obtained from the corresponding author upon request.

## Declarations

### Key References

$\bullet$ Hodnebrog, Ø., Myhre, G., Jouan, C., Andrews, T., Forster, P. M., Jia, H., Loeb, N. G., Olivié, D. J. L., Paynter, D., Quaas, J., Raghuraman, S. P., and Schulz, M.: Recent reductions in aerosol emissions have increased Earth’s energy imbalance, Communications Earth and Environment, 5, 1–9, https://doi.org/10.1038/s43247-024-01324-8, 2024. **This modeling study quantifies the relative contributions of GHGs and aerosols to the EEI trend, demonstrating that recent reductions in aerosols have contributed to increased heat in the Earth system.**

$\bullet$ Mülmenstädt, J., Salzmann, M., Kay, J. E., Zelinka, M. D., Ma, P. L., Nam, C., Kretzschmar, J., Hörnig, S., and Quaas, J.: An underestimated negative cloud feedback from cloud lifetime changes, Nature Climate Change, 11, 508–513, https://doi.org/10.1038/s41558-021-01038-1, 2021. **This study discusses biases in cloud phase feedback that are amplified by microphysical biases in models, and emphasizes the importance of improving cloud process representation through observational constraints.**

$\bullet$ Zelinka, M.D., Smith, C.J., Qin, Y., Taylor, K.E.: Comparison of methods to estimate aerosol effective radiative forcings in climate models. Atmospheric Chemistry and Physics 23, 8879–8898 (2023) **This study thoroughly documents the aerosol ERFs in CMIP models, quantifies how these are influenced by calculation methods, and examines the divergence among models by decomposing the ERFs into LW and SW components as well as microphysical contributions.**
